# Immunosuppression in down syndrome regression disorder: a prospective observational cohort study

**DOI:** 10.1093/braincomms/fcag203

**Published:** 2026-06-02

**Authors:** Jonathan D Santoro, Lilia Kazerooni, Maeve C Lucas, Mariam M Yousuf, Samuel T Otey, Samuel Bouchard, Kevin C Frost, Lina Nguyen, Nidhiben A Anadani, Asef Mahmud, Melanie A Manning, Angela L Rachubinski, Ryan M Kammeyer, Lina Patel, Lindsey E A Koster, Agnies M van Eeghen, Noemi A Spinazzi, Catherine Franklin, Eileen A Quinn, Joaquin M Espinosa, Michael S Rafii

**Affiliations:** Division of Neurology, Department of Pediatrics, Children’s Hospital Los Angeles, Los Angeles, CA 90027, USA; Department of Neurology, Keck School of Medicine of the University of Southern California, Los Angeles, CA 90027, USA; Division of Neurology, Department of Pediatrics, Children’s Hospital Los Angeles, Los Angeles, CA 90027, USA; Division of Neurology, Department of Pediatrics, Children’s Hospital Los Angeles, Los Angeles, CA 90027, USA; Division of Neurology, Department of Pediatrics, Children’s Hospital Los Angeles, Los Angeles, CA 90027, USA; Division of Neurology, Department of Pediatrics, Children’s Hospital Los Angeles, Los Angeles, CA 90027, USA; Division of Neurology, Department of Pediatrics, Children’s Hospital Los Angeles, Los Angeles, CA 90027, USA; Division of Neurology, Department of Pediatrics, Children’s Hospital Los Angeles, Los Angeles, CA 90027, USA; Division of Neurology, Department of Pediatrics, Children’s Hospital Los Angeles, Los Angeles, CA 90027, USA; Department of Neurology, University of Oklahoma Medical Center, Oklahoma City, OK 73104, USA; Advent Health, Orlando, FL 32803, USA; Department of Anatomic and Clinical Pathology, Stanford University School of Medicine, Palo Alto, CA 94305, USA; Linda Crnic Institute for Down Syndrome, Anschutz Medical Campus at the University of Colorado, Aurora, CO 80045, USA; Department of Neurology, Anschutz Medical Campus at the University of Colorado, Aurora, CO 80045, USA; Linda Crnic Institute for Down Syndrome, Anschutz Medical Campus at the University of Colorado, Aurora, CO 80045, USA; Amsterdam University Medical Center and Heeren Loo Care Group, Amsterdam 1105 AZ, Netherlands; Amsterdam University Medical Center and Heeren Loo Care Group, Amsterdam 1105 AZ, Netherlands; Department of Pediatrics, Specialized Children’s Hospital, New Brunswick, NJ 08901, USA; Mater Research Institute, The University of Queensland, Brisbane 4101, Australia; Department of Pediatrics, University of Toledo, Toledo, OH 43606, USA; Linda Crnic Institute for Down Syndrome, Anschutz Medical Campus at the University of Colorado, Aurora, CO 80045, USA; Alzheimer's Therapeutic Research Institute, University of Southern California, San Diego, CA 92121, USA

**Keywords:** down syndrome, rituximab, regression, mycophenolate mofetil, Janus kinase

## Abstract

Down syndrome regression disorder is a severe neuropsychiatric condition for which intravenous immunoglobulin offers partial benefit in many cases. The efficacy of second-line immunosuppressive therapies in those with partial responses to intravenous immunoglobulin (IVIg) remains unclear. This study sought to evaluate the comparative efficacy of B-cell depletion, Janus kinase inhibition, and mycophenolate mofetil as second-line immunosuppressive therapies in individuals with Down syndrome regression disorder. This multicenter, prospective observational cohort study included 126 individuals with Down syndrome regression disorder. Participants were aged 10–30 years and had demonstrated >50% improvement following IVIg on either the Bush-Francis Catatonia Rating Scale or the Neuropsychiatric Inventory Questionnaire, followed by second-line immunosuppression with one of three agents. Participants received B-cell depletion (rituximab or biosimilar; *n* = 63), Janus kinase inhibitors (tofacitinib or baricitinib; *n* = 34), or mycophenolate mofetil (*n* = 29). Treatments were assigned as part of clinical care and not randomized. The primary outcomes were change scores (Δ) on the Bush-Francis Catatonia Rating Scale and the Neuropsychiatric Inventory Questionnaire following immunosuppression. Secondary outcomes included treatment-emergent adverse event rates. All therapies produced symptomatic improvement; however, mean Δ Bush-Francis Catatonia Rating Scale and Δ Neuropsychiatric Inventory Questionnaire scores were greatest with B-cell depletion (mean [standard deviation] Δ Bush-Francis Catatonia Rating Scale: −9.6 [4.1]; Δ Neuropsychiatric Inventory Questionnaire: −16.5 [6.1]) compared with Janus kinase inhibition (Δ Bush-Francis Catatonia Rating Scale: −6.3 [5.0]; Δ Neuropsychiatric Inventory Questionnaire: −12.0 [7.2]) and mycophenolate mofetil (Δ Bush-Francis Catatonia Rating Scale: −3.0 [4.3]; Δ Neuropsychiatric Inventory Questionnaire: −5.7 [6.8]). One-way ANOVA showed significant between-group differences for both Bush-Francis Catatonia Rating Scale (*P* < 0.001) and Neuropsychiatric Inventory Questionnaire (*P* < 0.001). Post hoc Tukey tests revealed B-cell depletion to be significantly more effective than mycophenolate mofetil on both outcomes. Adverse event rates were lowest in the B-cell depletion group (2.40 events/patient) compared with Janus kinase inhibition (3.32) and mycophenolate mofetil (4.14) (overall *P* < 0.001). In individuals with Down syndrome regression disorder who partially respond to intravenous immunoglobulin, second-line immunosuppression with B-cell depletion was associated with the greatest clinical improvement and the most favorable safety profile.

## Teaser

Santoro *et al*. report that for individuals with Down Syndrome Regression Disorder who partially responded to intravenous immunoglobulin, B-cell depletion therapy is more effective and has fewer side effects than Janus kinase inhibition or mycophenolate mofetil. A longer period of illness before starting treatment is linked to a poorer response.

## Graphical Abstract

**Graphical Abstract:**
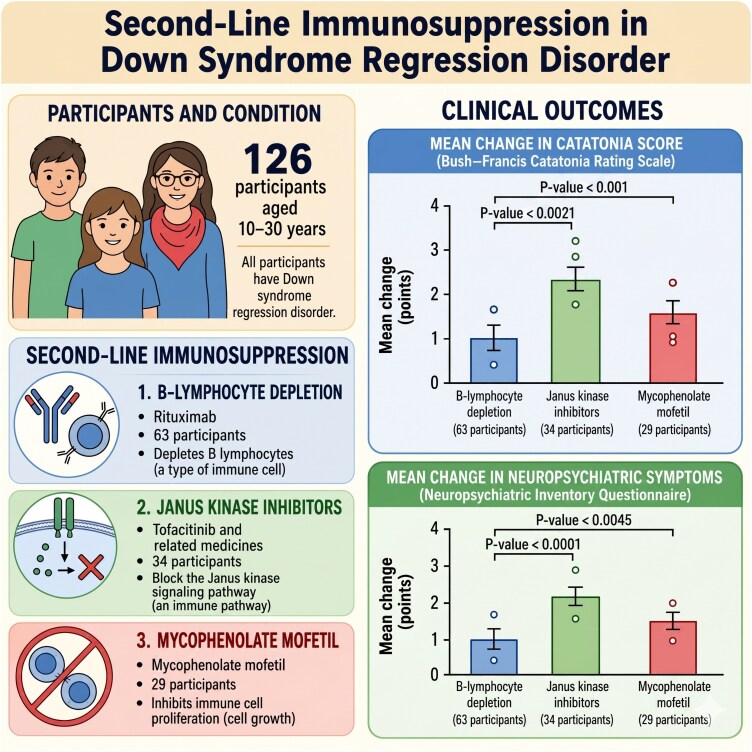

## Introduction

Individuals with Down syndrome (DS) are at risk for a variety of neurological and neuropsychiatric conditions across the lifespan.^1,2^ Down Syndrome Regression Disorder (DSRD) is an acute or subacute onset cognitive regression associated with apathy, avolition, bradykinesia, catatonia, delusions, hallucinations and other neuropsychiatric disturbances which most commonly develops in adolescence or early adulthood.^3-6^ This condition is severe, often causing significant distress for both the individual and caregivers.^7^

Individuals with DSRD have been treated with a variety of therapies including immunomodulation.^3,4,8,9^ Multiple studies have identified that individuals with DSRD are highly responsive to treatment with IVIg, which is both well tolerated and effective.^10^ Studies have also found that the presence of neurodiagnostic abnormalities on neuroimaging or lumbar punctures (LP) during the diagnostic evaluation predict higher likelihoods of response.^3,9,11^ In those individuals who are partially responsive to IVIg, the role of immunosuppressive agents is limited although reports of therapeutic benefit have been previously published.^3,9,12^

This prospective, observational study sought to assess the efficacy of immunosuppressive therapies as second line treatment in individuals with DSRD who were partially responsive to IVIg. In addition, the authors sought to identify factors that could influence the likelihood of response.

## Materials and methods

### Standard protocol approvals, registrations and patient consents

Institutional Review Board (IRB) approval was obtained for this study. Upon obtainment of consent of caregivers, and/or assent from participants, when possible, individuals were prospectively enrolled into an observational longitudinal database study. This study was not a randomized clinical trial and reports outcomes associated with treatment of patients as part of clinical care only.

### Participants and study design

Individuals were identified by having participated in either in-person or telehealth-based clinical consultation for DSRD at one of multiple sites between July 1, 2019, and June 30, 2025. Inclusion criteria included (i) age at onset of symptoms between 10–30 years, (ii) diagnosis of either possible or probable DSRD per consensus guidelines,^13^ (iii) completion of neurodiagnostic studies [EEG, MRI, and lumbar puncture (LP)] advised by the expert consensus guidelines, (iv) received IVIg previously and had a positive treatment response defined as >50% improvement on the Bush-Francis Catatonia Rating Scale (BFCRS) and/or the Neuropsychiatric Inventory Questionnaire (NPI-Q), (v) received treatment with IVIg for a period of one year, and (vi) been treated with an immunosuppressant (Appendix A) following incomplete response to IVIg. Age cut-offs were utilized to prevent possible diagnostic overlap with autism spectrum disorder and Alzheimer’s disease. Inclusion criteria 2 and 3 were adjudicated by an independent arbiter with no knowledge of the clinical history, diagnostic testing results, and/or treatment record retrospectively through chart review. Exclusion criteria included (i) age <10 years or >30 years at the time of symptom onset, (ii) complete response (100% back to baseline) in DSRD symptoms with IVIg or other therapies, (iii) active cardiac and/or pulmonary disease, (iv) frequent infection (defined as more than two infections requiring antibiotics or antivirals per year at any time in the past five years), (v) a history of neoplasia or receipt of chemotherapy or radiation, (vi) active or a history of epilepsy (excluding febrile seizure), (vii) use of electroconvulsive therapy (ECT) within the past five years, (viii) treatment immunotherapy not related to DSRD at the time of diagnosis and/or (ix) prior neurosurgical intervention of any type were excluded as well.

Demographic data, including medical/surgical history, and results of clinical and diagnostic investigations were collected from clinical documentation and entered into a de-identified form in a REDCap database. Individuals in this study were required to be evaluated on at least three occasions at intervals of no longer than 90 days by an unblinded clinical rater. All individuals in this study were assessed with the BFCRS and NPI-Q as part of each clinical visit. In addition, all individuals received a Clinical Global Impression—Severity Scale (CGI-S) at all visits and a Clinical Global Impression—Improvement Scale (CGI-I) at visits after therapeutics were initiated to assess global responses to interventions. These measures were chosen given their prior utilization in neuropsychiatric intervention trials^14^ and DSRD.^3,8^

### Definition of prior therapeutic response to IVIg

Clinical response was classified as a >50% improvement in at least one metric (BFCRS, NPI-Q, CGI-S and CGI-I) 24 weeks following commencement of IVIg.

### Determination of an abnormal neurodiagnostic study

Diagnostic abnormalities were determined to be abnormal using previously published definitions for DSRD (Appendix B).^9,11^ All cases where an abnormality was identified were confirmed by a blinded clinical adjudicator.

### Dosing of immunosuppression

Immunosuppressives were utilized as per previously published protocols.^3^ Patients were offered immunosuppression as part of clinical care, and treatment selection was determined by the managing clinician following multidisciplinary discussion that incorporated neurodiagnostic findings, comorbidities, and patient or caregiver preference. Decision-making regarding selection of immunosuppressants included reviewing neurodiagnostic data, patient/caregiver preference, and multidisciplinary case discussion when appropriate although no specific therapeutic algorithm was utilized. Importantly, treatment selection was non-randomized.

Dosing of immunosuppressives was standardized and can be found in Appendix A. Individuals could not be on IVIg at the same time as immunosuppressants with the final dose standardized to two weeks prior to the initiation of any of the immunosuppressant therapies. Individuals could be on other non-immunotherapeutic agents during the treatment, but dosing of therapies was required to be unchanged throughout the study period with the exception of IVIg which was administered two or more weeks prior to observation in the post-IVIg data sets. Individuals could not continue on IVIg during the immunosuppressive treatment period. All patients were treated for a period of at least 6 months unless there was intolerance of the immunosuppressive regimen. Clinical data endpoints were assessed between 4 and 6 months on treatment to allow sufficient time for all therapies to reach therapeutic effect.

### Adverse event monitoring

Although this study was not a clinical trial, manual record review was utilized to determine if adverse events (AEs) had occurred. Common Terminology Criteria for Adverse Events (CTCAE) version 5 nomenclatures and grading was applied to possible AEs identified on record review.

### Statistical analysis

Descriptive statistics were utilized to report demographic, clinical, and neurodiagnostic data. These data were subsequently compared between individuals receiving different immunosuppressive therapies using chi-square (χ^2^) or Fisher’s exact tests for categorical variables, and t-tests for continuous variables. For each treatment group, mean scores and standard errors of the mean (SEM) were calculated at each of the three timepoints (initial, post-IVIg and post-immunosupression). To evaluate differences in change scores (ΔBFCRS and ΔNPI-Q) across the three treatment groups, a one-way analysis of variance (ANOVA) was performed, followed by Tukey’s honestly significant difference (HSD) post hoc testing to identify pairwise differences between treatment groups. Group comparisons were performed at the post-immunosuppression timepoint to evaluate treatment-related differences. Independent two-sample *t* tests were conducted to compare: (i) B-cell depletion versus MMF and (ii) JAK inhibitor versus MMF. A two-sided *P*-value < 0.05 was considered statistically significant. No correction was applied for multiple comparisons given the exploratory design. A post-hoc Silverman’s test for multimodality was used to evaluate for skewed distribution in responses by therapy arm.

To control for potential confounding variables influencing treatment response, a multivariate logistic regression analysis was performed. The dependent variable was clinical response (defined as ≥50% improvement on the BFCRS or NPI-Q after immunosuppressive was administered). Independent variables included age at onset, sex, DSRD severity (possible versus probable), presence of neurodiagnostic abnormalities (EEG, MRI, LP), and treatment type (B-cell depletion, JAK inhibitor, or MMF). Odds ratios (OR) with 95% confidence intervals (CI) were calculated. Additionally, a multivariate general linear model (GLM) was used to assess predictors of change in continuous clinical outcome scores (ΔBFCRS and ΔNPI-Q). Model assumptions were assessed for collinearity, normality, and homoscedasticity. These models were designed to partially account for confounding by indication inherent in the non-randomized treatment assignment.

AE event rates were compared using Poisson regression with robust standard errors, and rate ratios (RRs) with 95% Wald CIs were reported. For binary outcomes (e.g. proportion of patients with ≥1 AE), we used χ^2^ tests of independence and pairwise comparisons via risk ratios (RRs) with 95% CIs. For AE subtype analyses, we aggregated events by system organ class and compared rates across arms using χ^2^ tests for rate homogeneity. Where AE types were exclusive to a single arm (e.g. infusion reactions), Fisher’s exact test or continuity-corrected χ^2^ was used as appropriate. A *P*-value < 0.05 was considered statistically significant.

### Data visualization

Descriptive data were visualized using bar graphs with treatment group–specific color coding. Error bars represent ±1 SEM and were displayed with capped whiskers. Jittered grey points were overlaid to illustrate individual-level variability. Asterisks were used to denote statistically significant pairwise comparisons at the post-immunosupressive timepoint. All figures were generated using Python (version 3.10) with Matplotlib and Seaborn libraries.

## Results

In total, 422 individuals were identified for inclusion. Of these, 47 (11.1%) were fully responsive to IVIg and 120 (28%) had a suboptimal response to IVIg resulting in exclusion from this study. A total of 255 (60%) individuals were determined to be partially responsive IVIg with a total of 127 (49.8%) who subsequently received immunosuppression with a defined therapeutic agent. During the 6-month follow-up period, no individuals discontinued their immunosuppressive regimen due to intolerability.

Clinical and demographic data are presented in Table 1. Similar to previously published studies, there was a mild over-representation of male participants in this cohort (69, 55%). Most participants met criteria for probable DSRD (94, 75%). The median ages at symptom onset, diagnosis, and first treatment were 17 (IQR: 13–20), 18 (IQR: 14–20), and 18 years (IQR: 14–21), respectively. Individuals in this cohort were initially treated with IVIg at a median age of 19 years (IQR 14–21) and continued on therapy for a median duration of 14 months (IQR: 12–16) prior to initiating immunosuppression. Neurodiagnostic abnormalities were present in many of the cohort including EEG (34, 27%), MRI (42, 33%), and LP (26, 21%) similar to previously published cohorts.^3,9^ Immunotherapeutics utilized were rituximab or biosimilar (63, 50%), tofacitinib (33, 26%), MMF (29, 23%), baricitinib (1, <1%), and azathioprine (1, <1%). Given the low utilization of both baricitinib and azathioprine, the former was grouped with tofacitinib and unified as a JAK inhibitor therapy category and the latter was removed from analysis due to dissimilar mechanism of action to other therapeutic categories.

**Table 1:** Clinical and demographic information (*n* = 126)

	*n* (%)
Sex	
* Male*	69 (55%)
* Female*	57 (45%)
Race	
* Caucasian*	91 (72%)
* Black/African-American*	18 (14%)
* Asian*	11 (9%)
* Other*	6 (5%)
Ethnicity	
* Hispanic*	51 (40%)
* Non-Hispanic*	75 (60%)
History of Autoimmune Disease	62 (49%)
History of Autism Spectrum Disorder	9 (7%)
Region of Primary Evaluation^a^	
* Northeast*	5 (4%)
* Midwest*	14 (11%)
* South*	6 (5%)
* West*	101 (80%)
Age at Symptom Onset (median, IQR)	17 (13–20)
Age at Diagnosis (median, IQR)	18 (14–20)
Age at First Treatment (median, IQR)	18 (14–20)
Age at Treatment with IVIg (median, IQR)	19 (14–21)
Duration on IVIg in Months (median, IQR)	14 (12–16)
DSRD Criteria	
* Possible*	32 (25%)
* Probable*	94 (75%)
DSRD Symptoms	
* Altered Mental Status or Behavioural Dysregulation*	113 (90%)
* Cognitive Decline*	109 (87%)
* Developmental Regression with or without New Autistic Features*	126 (100%)
* New Focal Neurological Deficits on Examination and/or Seizure*	14 (11%)
* Insomnia or Circadian Rhythm Disruption*	79 (63%)
* Language Deficits*	101 (80%)
* Movement Disorder*	111 (88%)
* Psychiatric Symptoms*	116 (92%)
Catatonia	93 (74%)
EEG Abnormal	34 (27%)
MRI Abnormal	42 (33%)
* T1 Abnormality*	0/42 (0%)
* T2 Signal Prolongation (>2 mm in white matter)*	10/42 (24%)
* GRE/SWI Signal Abnormality (bilateral basal ganglia)*	34/42 (81%)
* Multiple Abnormalities (T2* *+* *GRE/SWI)*	2/42 (5%)
Lumbar Puncture Abnormality	26 (21%)
* Pleocytosis (WBC > 5 cell/hpf)*	2/26 (8%)
* Elevated Total Protein*	9/26 (35%)
* Oligoclonal Bands*	7/26 (27%)
* IgG Index Elevation*	8/26 (31%)
* Neopterin*	2/23 (9%)
Multiple Neurodiagnostic Studies Abnormal	38 (30%)
Initial (Pre-IVIg) BFCRS (median, IQR)	22 (15–28)
Initial (Pre-IVIg) NPI-Q Total Score (median, IQR)	37 (30–45)
Immunosuppressive Received	
* Azathioprine*	1 (<1%)
* Baricitinib*	1 (<1%)
*Mycophenolate Mofetil*	29 (23%)
* Rituximab (or biosimilar)*	63 (50%)
* Tofacitinib*	33 (26%)
Concomitant Medications	
* Anticonvulsants (for mood stabilization)*	12 (10%)
* Antipsychotics* ^b^	18 (14%)
* Benzodiazepines*	69 (55%)
* Selective Serotonin Reuptake Inhibitors*	78 (62%)
* Tricyclic Antidepressants*	15 (12%)

BFCRS, Bush-Francis Catatonia Rating Scale; GRE, gradient echo; IQR, interquartile range; IVIg, Intravenous Immunoglobulin; JAK, Janus Kinase; NPI-Q, Neuropsychiatric Inventory Questionnaire; SWI, susceptibility weighted images; WBC, white blood cell.

^a^The Northeast Region includes Connecticut, Maine, Massachusetts, New Hampshire, New Jersey, New York, Pennsylvania, Rhode Island, and Vermont; the Midwest Region includes Illinois, Indiana, Iowa, Kansas, Michigan, Minnesota, Missouri, Nebraska, North Dakota, Ohio, South Dakota, and Wisconsin; the South Region includes Alabama, Arkansas, Delaware, Florida, Georgia, Kentucky, Louisiana, Maryland, Mississippi, North Carolina, Oklahoma, South Carolina, Tennessee, Texas, Virginia, Washington D.C., and West Virginia; the West Region includes Alaska, Arizona, California, Colorado, Hawaii, Idaho, Montana, Nevada, New Mexico, Oregon, Utah, Washington, and Wyoming.

^b^Includes typical and atypical antipsychotics (e.g. aripiprazole, haloperidol, quetiapine, and risperidone).

**Figure 1:**
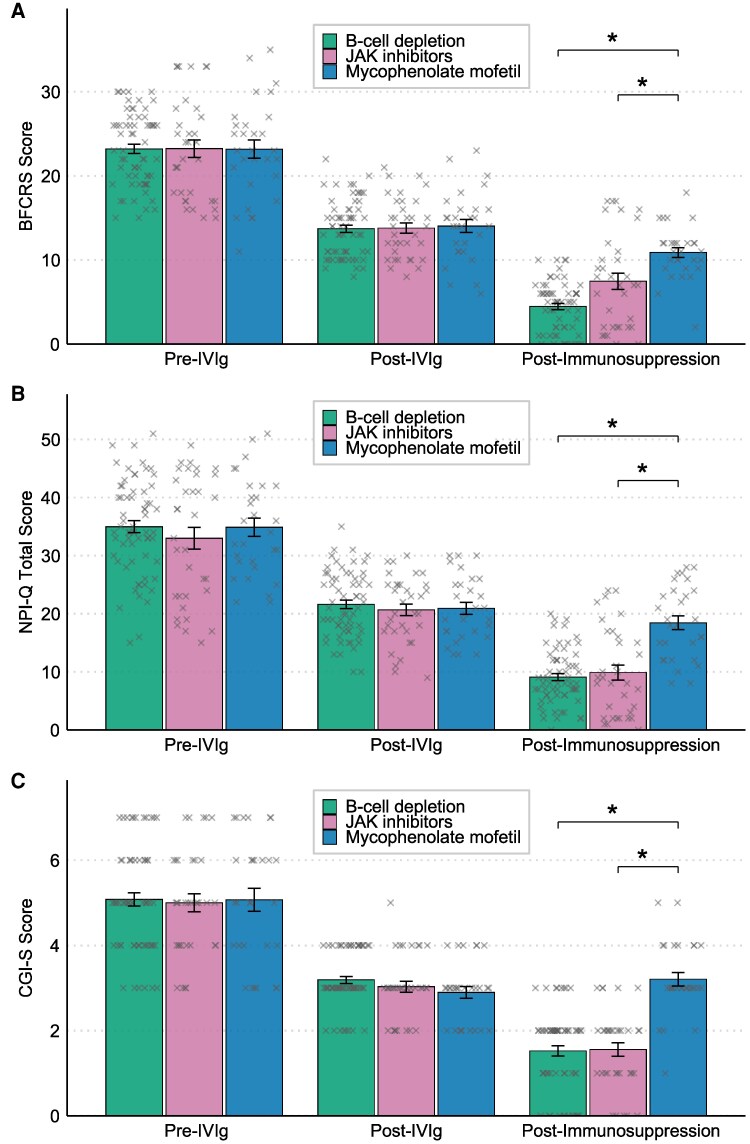
**Effects of immunosuppression on BFCRS.** (**A**), NPI-Q (**B**), and CGI-S (**C**) scores at baseline (pre-IVIg), after IVIg therapy (post-IVIg), and following second-line immunosuppression (post-immunosuppression). Bars represent mean ± standard error of mean with jittered individual data points representing individual participants with DSRD (B-cell depletion *n* = 63, JAK inhibitor *n* = 34, mycophenolate mofetil *n* = 29). Between-group comparisons at the post-immunosuppression timepoint were performed using one-way ANOVA followed by Tukey HSD post-hoc testing. Asterisks indicate statistically significant pairwise comparisons (*P < 0.05). BFCRS, Bush-Francis Catatonia Rating Scale; CGI-S, Clinical Global Impression—Severity Scale; IVIg, Intravenous Immunoglobulin; JAK, Janus Kinase; NPI-Q, Neuropsychiatric Inventory Questionnaire.

Therapeutic responses over time are presented in Fig. 1 and Table 2. The mean BFCRS scores declined over time in all treatment groups, indicating clinical improvement. By the post-immunosuppression timepoint, individuals receiving B-cell depletion showed the most substantial reduction in catatonia severity (mean score ± SEM: 4.2 ± 1.1), followed by the JAK inhibitor group (7.6 ± 1.4), and the MMF group (11.3 ± 1.6). Group comparisons at the post-immunosuppression timepoint revealed a statistically significant difference between B-cell depletion and MMF (*P* = 0.04), as well as between JAK inhibition and MMF (*P* < 0.03). Similarly, NPI-Q total scores decreased across all treatment arms, consistent with an improvement in behavioural and psychiatric symptoms. At the post-immunosuppression timepoint, individuals treated with B-cell depletion demonstrated the greatest reduction (mean score ± SEM: 5.1 ± 1.3), followed by JAK inhibitor (8.8 ± 1.5), and mycophenolate mofetil (13.9 ± 1.9). Statistically significant differences were observed between B-cell depletion and MMF (*P* = 0.02), as well as JAK inhibitor and MMF (*P* = 0.03).

**Table 2:** Therapeutic responses to immunosuppression

	B-Cell Depletion *n* = *63*	JAK Inhibitors *n* = *34*	Mycophenolate Mofetil *n* = *29*
Initial Score (Pre-IVIg)			
BFCRS	22 (19–26)	23 (17–28)	23 (19.5–26.5)
NPI-Q	34 (29–41)	35 (23–44)	34 (28.5–42)
CGI-S	5 (4–6)	5 (4–6)	5 (4–6)
Post-IVIg Score			
BFCRS	14 (11–17)	13.5 (10–16.25)	14 (11–16)
NPI-Q	21 (17–26)	21 (17–26)	20 (16.5–25.5)
CGI-S	3 (3–4)	3 (2.75–3.25)	3 (2–3)
CGI-I	2 (2–3)	2 (2–3)	2 (2–3)
Post-Immune Suppression Score			
BFCRS	5 (2–7)	7 (2–11.25)	11 (9–12)
NPI-Q	8 (6–12)	8.5 (2–16.25)	18 (12–24)
CGI-S	2 (1–2)	2 (1–2)	3 (3–4)
CGI-I	2 (1–2)	2 (2–3)	4 (3–5)

BFCRS, Bush-Francis Catatonia Rating Scale; CGI-I, Clinical Global Impression—Improvement Scale; CGI-S, Clinical Global Impression—Severity Score; NPI-Q, Neuropsychiatric Inventory Questionnaire.

All values presented as median (interquartile range).

Within-group analyses demonstrated significant improvements in CGI-S scores across all three treatment cohorts (Table 2 and Fig. 1C). Individuals treated with B-cell depletion and JAK inhibitors had a mean reduction of 3.55 and 3.43-point reduction, on the CGI-S, respectively. This was statistically significant compared with the 1.84-point increase observed in those receiving MMF at the post-immunosuppression time point (*P* < 0.001, 95% CI: 2.31–6.42 B-cell depletion v MMF and *P* < 0.001, 95% CI: 1.79–5.23 JAK inhibitor v. MMF). There was no statistical difference between the post-IVIg and post-immunosuppression with MMF time points (*P* = 0.07, 95% CI: −0.71–0.03). There were no statistically significant differences on CGI-I between the post-IVIg and post-immunosuppression time points for any treatment group.

**Figure 2:**
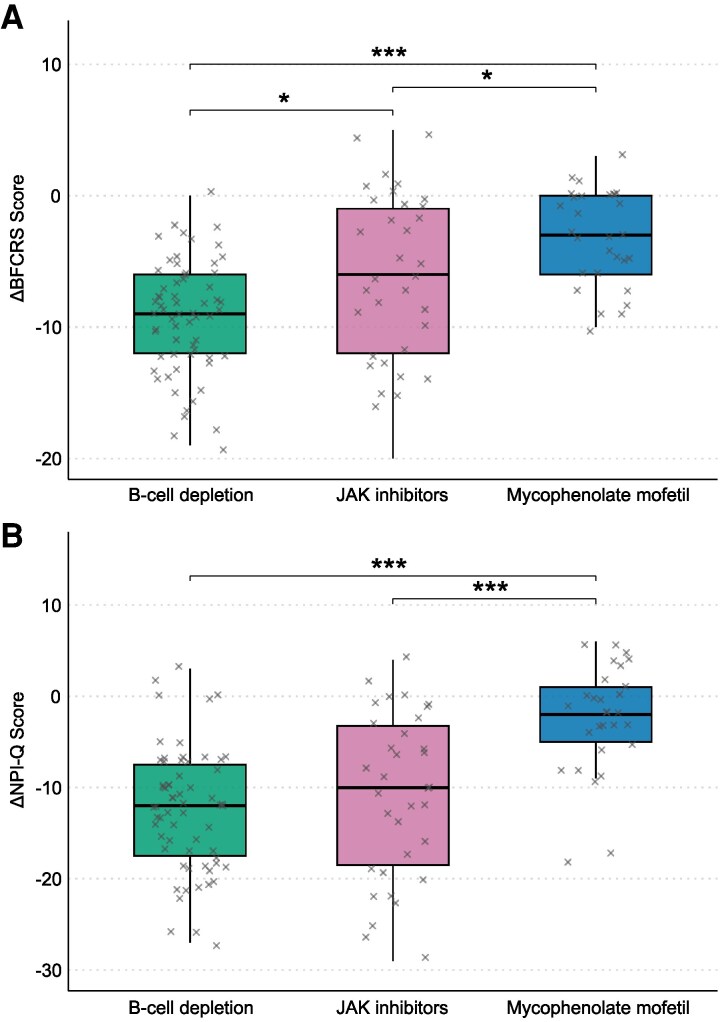
**Change scores (Δ) in BFCRS.** (**A**) NPI-Q (**B**) following second-line immunosuppression by treatment group. Negative values indicate clinical improvement. Bars represent mean ± standard error of mean and grey data points represent individual participant change scores (B-cell depletion *n* = 63, JAK inhibitor *n* = 34, mycophenolate mofetil *n* = 29). Group differences were evaluated using one-way analysis of variance (ANOVA) with Tukey HSD post-hoc testing (ΔBFCRS: F(2, 123) = 16.54, *P* < 0.001; ΔNPI-Q: F(2, 121) = 19.67, *P* < 0.0001). Asterisks denote statistically significant pairwise comparisons (*P < 0.05; ***P < 0.001). BFCRS, Bush-Francis Catatonia Rating Scale; IVIg, Intravenous Immunoglobulin; JAK, Janus Kinase; NPI-Q, Neuropsychiatric Inventory Questionnaire.

A one-way ANOVA was conducted to evaluate change (Δ) on both the BFCRS and NPI-Q among each treatment group. The analysis revealed a statistically significant overall effect of treatment group on ΔBFCRS scores, F(2, 123) = 16.54, *P* < 0.001 (Fig. 2A) and on ΔNPI-Q scores, F(2, 121) = 19.67, *P* < 0.0001 (Fig. 2B). Post hoc pairwise comparisons using a Tukey HSD test demonstrated that B-cell depletion resulted in significantly greater improvements compared with MMF (mean difference BFCRS = 6.12, *P* < 0.001 95% CI: 3.55, 8.68; mean difference NPI-Q = 10.04, *P* < 0.001, 95% CI: 6.20, 13.89). B-cell depletion resulted in a significantly greater improvement compared with JAK inhibition on the BFCRS (mean difference = 2.93, *P* = 0.014, 95% CI: 0.50, 5.36) but not the NPI-Q (mean difference = 1.73, *P* = 0.50, 95% CI: −1.92, 5.38). Additionally, JAK inhibition showed a significantly greater effect than MMF (mean difference BFCRS = 3.19, *P* = 0.027, 95% CI: 0.30, 6.07; mean difference NPI-Q = 8.31, *P* < 0.001, 95% CI: 3.98, 12.64).

**Figure 3:**
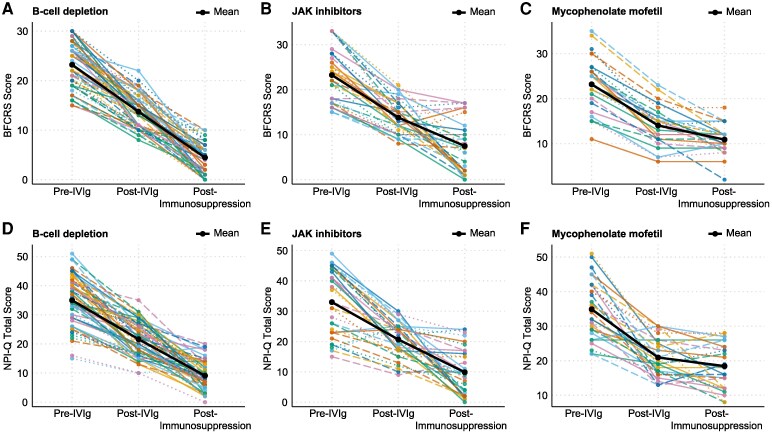
**Individual patient trajectories (‘spaghetti plots’) demonstrating longitudinal changes in BFCRS (A–C) and NPI-Q (D–F) scores across treatment groups.** Each line represents a single participant with DSRD treated with B-cell depletion (A, D; *n* = 63), JAK inhibition (B, E; *n* = 34), or mycophenolate mofetil (C, F; *n* = 29). These plots illustrate within-individual variability in treatment response across the observational treatment period. BFCRS, Bush-Francis Catatonia Rating Scale; CGI-S, Clinical Global Impression—Severity Scale; IVIg, Intravenous Immunoglobulin; JAK, Janus Kinase; NPI-Q, Neuropsychiatric Inventory Questionnaire.

Individual patient trajectories based on therapeutic intervention are displayed in Fig. 3. Notably, this revealed that while responses to B-Cell depletion (Fig. 3A and D) and MMF (Fig. 3C and F) were relatively homogeneous, responses to JAK inhibitors were more variable (Fig. 3B and E). Silverman’s test for multimodality revealed that B-cell depletion (*P* = 0.99, *P* = 0.90) and MMF (*P* = 0.97, *P* = 0.56) were unimodal whereas JAK inhibitors (*P* = 0.04, *P* = 0.03) were bimodal on BFCRS and NPI-Q, respectively.

The likelihood of therapeutic responses based on neurodiagnostic studies abnormalities is presented in Table 3. Notably, individuals receiving B-cell depletion were significantly more likely to respond to immunosuppression if they had abnormal LP results (OR: 8.66, *P* < 0.001, 95% CI: 2.37–9.44) whereas individuals receiving JAK inhibitors were more likely to respond if they had an abnormal MRI (OR: 3.98, *P* < 0.001, 95% CI: 1.27–6.41).

**Table 3:** Multivariate logistic regression predicting therapeutic response to immunosuppression based on clinical and neurodiagnostic abnormalities

	aOR	*P* value	95% CI
Age at Symptom Onset	0.97	0.43	0.89–1.09
Sex	1.14	0.67	0.62–2.13
Race (Caucasian Ref)			
* Black/African-American*	0.91	0.84	0.38–2.18
* Asian*	1.06	0.91	0.41–2.74
* Other*	0.98	0.97	0.26–3.61
DSRD Diagnosis	1.33	0.41	0.67–2.65
Duration of Illness (months)	**0**.**54**	**0**.**003**	**0.64–0.89**
B-Cell Depletion			
* EEG*	0.89	0.77	0.31–3.48
* MRI*	1.36	0.21	0.44–4.91
* Lumbar Puncture*	**8**.**66**	**<0**.**001**	**2.37–9.44**
JAK Inhibitor			
* EEG*	1.07	0.85	0.25–2.31
* MRI*	**3**.**98**	**<0**.**001**	**1.27–6.41**
* Lumbar Puncture*	*1*.*54*	*0*.*09*	*0.87–3.22*
Mycophenolate Mofetil			
* EEG*	1.11	0.63	0.49–3.27
* MRI*	0.92	0.73	0.34–5.71
* Lumbar Puncture*	1.29	0.19	0.69–3.08

aOR, Adjusted odds ratio; CI, confidence interval; EEG, electroencephalogram.

Bolded items indicate *P* value < 0.001. Italicized items indicate trending *P*-value (0.05–0.10). Bolded items indicate statistical significance of *P* < 0.05. Therapeutic response as >50% improvement on either the BFCRS and/or NPI-Q following administration of immunosuppression.

To further assess predictors of treatment response, multivariate analyses were conducted (Table 3). Neither age at symptom onset, sex, race, nor DSRD severity was significantly associated with response. A history of prior autoimmune disease did lead to a higher likelihood of response to JAK inhibitors (OR: 2.31, *P* = 0.03, 95% CI: 1.19–3.12) but not B-cell depletion (*P* = 0.64) or MMF (*P* = 0.79). A longer duration of illness prior to immunosuppression was independently associated with a lower likelihood of response to all immunosuppressive therapies (OR = 0.54, *P* = 0.003, 95% CI: 0.64–0.89). Similarly, a general linear model examining the magnitude of symptom change, duration of illness remained a significant negative predictor of ΔBFCRS (*β* = −0.42, *P* = 0.002) and ΔNPI-Q (*β* = −0.58, *P* = 0.003), indicating that longer-standing symptoms were associated with less robust improvement (Table 4).

**Table 4:** General linear model predicting change in clinical endpoints following immunosuppression on BFCRS and NPI-Q

Predictor	*β* (ΔBFCRS)	95% CI	*P*-value	*β* (ΔNPI-Q)	95% CI	*P*-value
Age at Symptom Onset (years)	−0.14	−0.39–0.11	0.27	−0.08	−0.42–0.25	0.64
Sex	0.52	−0.14–2.19	0.53	1.02	−1.68–3.72	0.45
Race (Caucasian ref.)						
Black/African-American	−0.37	−2.95–2.20	0.77	−1.41	−5.49–2.66	0.49
Asian	0.69	−2.15–3.53	0.63	0.42	−3.92–4.75	0.85
Other	1.01	−3.66–5.68	0.67	−0.11	−6.14–5.92	0.97
DSRD Severity	0.93	−0.81–2.67	0.29	1.65	−1.33–4.63	0.27
Duration of Illness (months)	−0.42	−0.68–−0.15	0.002	−0.58	−0.96–−0.21	0.003

*β*, unstandardized regression coefficient; BFCRS, Bush-Francis Catatonia Rating Scale; CI, confidence interval; LP, lumbar puncture; MMF, mycophenolate mofetil; MRI, magnetic resonance imaging; NPI-Q, Neuropsychiatric Inventory Questionnaire.

Negative *β* coefficients represent greater symptom improvement (lower scores). Bold indicates statistical significance (*P* < 0.05).

No individuals in this study discontinued immunosuppressive therapy due to intolerability. A review of reported adverse events is presented in Table 5. Across immunosuppressive therapies, significant differences were observed in the overall number and type of AEs. Patients receiving B-cell depletion had the lowest AE rate at 2.40 events per patient, compared with 3.32 for JAK inhibitors and 4.14 for MMF (χ^2^(2) = 20.92, *P* = 2.9 × 10^−5^). Pairwise comparisons showed that B-cell depletion was associated with significantly fewer AEs than both JAK inhibitors [rate ratio (RR) 0.72, *P* = 0.01, 95% CI 0.57–0.92] and MMF (RR 0.58, *P* < 0.001, 95% CI 0.46–0.74). However, the proportion of individuals with at least one AE did not differ significantly between groups (χ^2^(2) = 2.24, *P* = 0.33). Specific AE types varied by treatment: gastrointestinal events were significantly more frequent in the JAK inhibitors (*P* < 0.001, RR: 0.13–0.54, 95% CI: 0.13–0.54) and MMF (*P* < 0.001, RR: 0.18, 95% CI: 0.09–0.40) groups compared with B-cell depletion. Metabolic events (hyperlipidemia/hyperglycemia) were more common in individuals treated with JAK inhibitors (*P* < 0.001, RR 0.060, 95% CI 0.0076–0.47) and MMF (*P* < 0.001, RR: 0.03, 95% CI: 0.01–0.27) compared with B-cell depletion. Rash was more frequent with JAK inhibitors than with B-cell therapy (*P* = 0.02, RR 0.29, 95% CI 0.11–0.8). No significant differences were found in nervous system, cardiovascular, hematologic, infectious, or general symptom AEs.

**Table 5:** Adverse events reported by treatment arm

	B-Cell Depletion (*n* = 63)				JAK Inhibitor (*n* = 34)				Mycophenolate Mofetil (*n* = 29)			
CTAEC 5 Grade	1	2	3	All	1	2	3	All	1	2	3	All
Total AE	81 (54%)	62 (41%)	8 (5%)	151	50 (44%)	56 (50%)	7 (6%)	113	68 (57%)	47 (39%)	5 (4%)	120
Total Patients with AE	17 (50%)	21 (62%)	2 (6%)	34 (54%)	13 (59%)	15 (68%)	3 (14%)	22 (65%)	13 (65%)	7 (35%)	2 (10%)	20 (69%)
Infusion Reaction	8 (57%)	5 (36%)	1 (7%)	14 (22%)	-	-	-	-	-	-	-	-
Infection												
* Serious Infection* ^a^	0 (0%)	0 (0%)	1 (100%)	1 (2%)	0 (0%)	0 (0%)	1 (100%)	1 (3%)	0 (0%)	0 (0%)	0 (0%)	0 (0%)
* Pharyngitis*	-	3 (100%)	0 (0%)	3 (5%)	-	5 (100%)	0 (0%)	5 (15%)	-	5 (100%)	0 (0%)	5 (17%)
* URI*	-	11 (92%)	1 (8%)	12 (19%)	-	8 (89%)	1 (11%)	9 (26%)	-	7 (100%)	0 (0%)	7 (24%)
* Lung Infection*	-	1 (100%)	0 (0%)	1 (2%)	-	0 (0%)	0 (0%)	0 (0%)	-	0 (0%)	0 (0%)	0 (0%)
* Nail Infection*	1 (100%)	0 (0%)	0 (0%)	1 (2%)	0 (0%)	1 (100%)	0 (0%)	1 (3%)	0 (0%)	0 (0%)	0 (0%)	0 (0%)
* Otitis Media*	-	1 (100%)	0 (0%)	1 (2%)	-	2 (100%)	0 (0%)	2 (6%)	-	3 (100%)	0 (0%)	3 (10%)
Blood/Lymphatic												
* Anemia*	0 (0%)	1 (100%)	0 (0%)	1 (2%)	1 (50%)	1 (50%)	0 (0%)	2 (6%)	0 (0%)	0 (0%)	0 (0%)	0 (0%)
* Leukopenia*	6 (40%)	2 (20%)	2 (20%)	10 (16%)	1 (25%)	2 (50%)	1 (25%)	4 (12%)	1 (33%)	2 (67%)	0 (0%)	3 (10%)
* Lymphopenia*	12 (50%)	10 (42%)	2 (8%)	24 (38%)	0 (0%)	3 (75%)	1 (25%)	4 (12%)	1 (33%)	1 (33%)	1 (33%)	3 (10%)
* Neutropenia*	4 (57%)	3 (43%)	0 (0%)	7 (11%)	3 (60%)	1 (20%)	1 (20%)	5 (15%)	0 (0%)	1 (50%)	1 (50%)	2 (7%)
* Thrombocytopenia*	0 (0%)	0 (0%)	0 (0%)	0 (0%)	0 (0%)	1 (100%)	0 (0%)	1 (3%)	1 (100%)	0 (0%)	0 (0%)	1 (3%)
Cardiovascular												
* Hypotension*	7 (50%)	6 (43%)	1 (7%)	14 (22%)	3 (43%)	3 (43%)	1 (14%)	7 (21%)	2 (100%)	0 (0%)	0 (0%)	2 (7%)
* Hypertension*	0 (0%)	1 (100%)	0 (0%)	1 (2%)	1 (33%)	2 (67%)	0 (0%)	3 (9%)	1 (100%)	0 (0%)	0 (0%)	1 (3%)
* Chest Pain*	1 (50%)	1 (50%)	0 (0%)	2 (3%)	0 (0%)	0 (0%)	0 (0%)	0 (0%)	1 (100%)	0 (0%)	0 (0%)	1 (3%)
Gastrointestinal												
* Nausea/Abd Pain*	7 (87%)	1 (13%)	0 (0%)	8 (13%)	7 (64%)	4 (36%)	0 (0%)	11 (32%)	9 (64%)	4 (29%)	1 (7%)	14 (48%)
* Vomiting (Emesis)*	2 (67%)	1 (33%)	0 (0%)	3 (5%)	3 (50%)	3 (50%)	0 (0%)	6 (18%)	2 (40%)	3 (60%)	0 (0%)	5 (17%)
* Diarrhea*	1 (100%)	0 (0%)	0 (0%)	1 (2%)	4 (57%)	3 (43%)	0 (0%)	7 (21%)	6 (54%)	4 (36%)	1 (9%)	11 (38%)
Metabolic												
* Elevated Cholesterol*	0 (0%)	0 (0%)	0 (0%)	0 (0%)	4 (57%)	3 (43%)	0 (0%)	7 (21%)	6 (86%)	1 (14%)	0 (0%)	7 (24%)
* Hyperglycemia*	1 (100%)	0 (0%)	0 (0%)	1 (2%)	2 (100%)	0 (0%)	0 (0%)	2 (6%)	7 (100%)	0 (0%)	0 (0%)	7 (24%)
Nervous System												
* Headache*	8 (80%)	2 (20%)	0 (0%)	10 (16%)	5 (87%)	1 (17%)	0 (0%)	6 (18%)	3 (75%)	1 (25%)	0 (0%)	4 (14%)
* Tremor*	0 (0%)	1 (100%)	0 (0%)	1 (2%)	1 (100%)	0 (0%)	0 (0%)	1 (3%)	5 (63%)	3 (37%)	0 (0%)	8 (28%)
Respiratory												
* Cough*	5 (100%)	0 (0%)	0 (0%)	5 (8%)	3 (100%)	0 (0%)	0 (0%)	3 (9%)	7 (78%)	2 (22%)	0 (0%)	9 (31%)
* Dyspnea*	0 (0%)	0 (0%)	0 (0%)	0 (0%)	0 (0%)	0 (0%)	0 (0%)	0 (0%)	0 (0%)	1 (100%)	0 (0%)	1 (3%)
Skin/Subcutaneous												
* Rash*	3 (50%)	3 (50%)	0 (0%)	6 (10%)	6 (55%)	5 (45%)	0 (0%)	11 (32%)	6 (75%)	2 (25%)	0 (0%)	8 (28%)
General												
* Chills/Rigors*	0 (0%)	1 (100%)	0 (0%)	1 (2%)	0 (0%)	0 (0%)	0 (0%)	0 (0%)	0 (0%)	1 (100%)	0 (0%)	1 (3%)
* Fatigue*	11 (73%)	4 (27%)	0 (0%)	15 (24%)	2 (25%)	5 (63%)	1 (13%)	8 (24%)	5 (56%)	3 (33%)	1 (11%)	9 (31%)
* Fever*	5 (83%)	1 (17%)	0 (0%)	6 (9%)	2 (50%)	2 (50%)	0 (0%)	4 (12%)	3 (60%)	2 (40%)	0 (0%)	5 (17%)
* Edema*	0 (0%)	2 (100%)	0 (0%)	2 (3%)	0 (0%)	1 (100%)	0 (0%)	1 (3%)	0 (0%)	0 (0%)	0 (0%)	0 (0%)
* Pain*	0 (0%)	1 (100%)	0 (0%)	1 (2%)	2 (100%)	0 (0%)	0 (0%)	2 (6%)	1 (50%)	1 (50%)	0 (0%)	2 (7%)

^a^Serious infection indicates need for emergency room visit, hospitalization and/or antibiotics, upper respiratory tract infection (URI).

Data on continuation of treatment in the post-study period was available for 78 individuals at one year (*n* = 31 b-cell depletion, *n* = 24 JAK inhibitors, *n* = 23 MMF). Among those receiving these therapies, 90% (28/31), 71% (17/24) and 65% (15/23) remained on their original therapy, respectively.

## Discussion

This study identified that immunosuppression was effective in managing DSRD in individuals who had previously partially responded to IVIg. Among the therapies, MMF was the least effective across all testing metrics whereas both B-cell depletion and JAK inhibition showed significant clinical benefits. While the response to B-cell depletion was consistent among individuals, the response to JAK inhibitors varied, with a notable distinction between responders and non-responders.

Reports on the use of immunosuppressive therapies is limited in individuals with DSRD although this study provided data consistent with prior reports.^3,12,15^ Notably, this data set identified that both B-cell depletion and JAK inhibition were more likely to be therapeutically beneficial for individuals as compared to MMF. While MMF was beneficial for some individuals, the majority had marginal clinical gain. This has been observed in some prior studies although the broad mechanism of this therapeutic may explain some of the heterogenous responses.^3^ In addition, this lack of observed clinical benefit may have been related to the lower rate of neurodiagnostic abnormalities present in those receiving MMF which could have either made them ineligible for ‘higher efficacy’ treatments or predisposed them to be less likely to respond to immunotherapy overall.

Multiple studies have demonstrated that abnormal neurodiagnostic studies are associated with a significantly higher rate of response to immunosuppression.^3,8,9,11^ These findings have been interpreted as evidence of active neuroinflammatory processes in a subset of individuals with the disorder. In the present cohort, similar patterns were observed, further supporting the potential utility of neurodiagnostic findings as biomarkers to identify individuals most likely to benefit from immunomodulatory therapy. This was evident in our cohort as well where individuals with abnormal neurodiagnostics were more likely to respond to IVIg initially and to subsequent immunosuppressive therapies. In our study, the only statistically significant drivers of treatment response were neuroimaging and LP abnormalities. This supported our rationale for use of B-cell depletion (abnormal CSF consistent with increased intrathecal antibody synthesis)^16^ and JAK inhibition (abnormal neuroimaging consistent with upregulated interferon response).^11,17^ This indicates that these neurodiagnostic abnormalities may support differentiated immunosuppressive therapy selection in individuals with DSRD. However, multivariate analysis identified that longer duration of symptoms negatively influenced the likelihood of response to all second line immunotherapies and as such must be a consideration when evaluating the risks and benefits of treatment with individuals and caregivers.

Safety profiles across all three immunosuppressive therapies revealed that treatments were well tolerated, with very few grade III AEs and no grade IV or V events. B-cell depletion was associated with greater clinical improvement and a favorable safety profile in this observational cohort. In contrast, JAK inhibitors and MMF were associated with higher rates of gastrointestinal, metabolic, and dermatologic adverse events although all AEs match previously published literature.^18-22^ While no individuals discontinued therapy due to intolerability, the frequency and system involvement of AEs varied substantially, underscoring the importance of aligning treatment selection with patient-specific risk factors. Importantly, the proportion of individuals experiencing at least one AE did not significantly differ between groups, suggesting that while AEs are common across all therapies, their severity and clinical impact may differ. These data suggest that B-cell depletion may offer both efficacy and safety advantages in appropriately selected patients although prospective trials with formal safety monitoring are needed to confirm these findings and refine risk–benefit stratification in DSRD.

The unique immunologic mechanisms for DSRD remain under active investigation. Previous studies have demonstrated potential roles for upregulated interferon responses^15,23-25^ and break down of the blood-brain-barrier, both of which may be factors influencing the development of the disease.^26^ JAK inhibition is currently under investigation not only for DSRD, but also for other systemic disorders associated with DS, due to its role in modulating interferon signalling and the JAK/STAT cascade.^27^ That said, interferon-mediated immune activation is complex, resulting in the potential for multiple unique immunotherapeutic targeting opportunities.^28-33^ One such pathway, the interferon-mediated induction of autoreactive B-cells, may partially explain why B-cell depletion was therapeutically beneficial in this cohort.^34^ Similarly, enhanced dendritic cell activation with antigen presentation and/or breakdown of B-cell tolerance could all potentially induce dysregulation in B-cell lines leading to autoantibody production.^35,36^ This is in line clinically with observed overproduction of autoantibodies in individuals with DS without regression or known neurological diseases, indicating a predisposition for errant B-cell mediated processes.^37^ Although DS is phenotypically and immunologically unique, the autoimmune and autoinflammatory diseases encountered in this condition may not be entirely dissimilar from other systemic conditions such as systemic lupus erythematosus or multiple sclerosis where multiple therapies with unique mechanisms of action are beneficial for the same clinical syndrome. Of note, a prior history of autoimmune disease was associated with greater responsiveness to JAK inhibition but not B-cell depletion. This may reflect the interferon-driven immune dysregulation characteristic of DS where therapies targeting upstream cytokine signalling such as JAK inhibitors may have broader effects than B-cell–directed therapies. Conversely, individuals with intrathecal immune activation (e.g. CSF abnormalities) may have disease mechanisms more closely related to B-cell activity, potentially explaining the observed association with response to B-cell depletion, although additional study is needed.

This study is not without limitations. These include severity bias, ascertainment bias, and selection bias, as the centers involved in this study are those evaluating high numbers of individuals with DSRD with capability to perform neurodiagnostic work up and therapeutic intervention with immunosuppression. The age range of 10–30 years was selected to improve diagnostic specificity and reduce potential overlap with autism spectrum disorder–related regression in younger children and Alzheimer-related cognitive decline in older individuals with DS. As such, the results of this study may not be directly generalizable to all individuals with DSRD, particularly those outside of the predefined age range. Similarly, the therapeutic approaches to immunosuppression, while previously published, were narrow and guided by the managing clinician. In this regard, individuals who were complete non-responders to IVIg were also excluded. While this was done for safety purposes to minimize exposure to higher risk immunomodulation, it presumably biased the sample towards individuals more likely to respond to immunosuppression. Further, individuals included could have had IVIg up to 2 weeks prior to the observational study period window which may have meant that some clinical benefit from IVIg may have been present in the early phases of evaluation. The effect of IVIg typically is out of the system by 8 weeks and as such this would not be anticipated to cause endpoint interpretation difficulties. Additionally, the clinical improvement metrics (BFCRS, NPI-Q, CGI-S, CGI-I) are not validated in individuals with DSRD or DS but are the most previously reported metrics to monitor disease activity. In addition, the NPI-Q, CGI-S and CGI-I rely on proxy report which may impose additional biases. Patients were on a variety of different non-immunotherapy symptomatic management protocols during the observational period, both regarding specific medications and dosing regimens. As such, sub-analysis was not possible due to being underpowered. In addition, systemic immune and inflammatory biomarkers (e.g. lymphocyte subsets, immunoglobulin levels, and circulating cytokines) were not collected in a standardized manner across participating sites, limiting our ability to correlate immunologic changes with treatment response. Prospective studies incorporating longitudinal immune profiling may provide additional mechanistic insight into therapeutic responsiveness in DSRD. Importantly, this study utilized a variable set of endpoints for documentation of clinical improvement which were not strict and may have overestimated clinical improvement. Most importantly, this study was not randomized or blinded which introduces selection bias and limits the ability to discern which of these therapies is categorically most beneficial for all individuals with DSRD. Patients without clear neurodiagnostic abnormalities or those in whom higher-intensity immunosuppression was not favored were more commonly treated with mycophenolate mofetil. Because these clinical factors influenced treatment selection, confounding by indication is possible. However, these real-world treatment patterns may provide useful insights for the design of future randomized trials that stratify patients according to neurodiagnostic or immunologic features. Therefore, the observed differences between therapeutic groups should be interpreted as hypothesis-generating rather than definitive evidence of comparative efficacy.

In summary, this study identified that individuals with DSRD who have a partial therapeutic response to IVIg may derive additional benefit from immunosuppression. Identification and categorization of neurodiagnostic abnormalities may be useful in guiding treatment selection. Double-blind, randomized clinical trials are still needed in this space to verify the findings from this study.

## Supplementary Material

fcag203_Supplementary_Data

## Data Availability

Anonymized data are available to qualified researchers upon request to the corresponding author and IRB approval.
