# Supplementary material for: Immunosuppression in down syndrome regression disorder: a prospective observational cohort study
Source: Brain Commun. 2026 Jun 2;8(3):fcag203. doi: 10.1093/braincomms/fcag203 (PMC13253568; doi:10.1093/braincomms/fcag203)
Supplement: fcag203_Supplementary_Data [file fcag203_supplementary_data.docx]

**Appendix A:** Immunosuppressants Studied

1. Azathioprine
   1. Goal dose 3 mg/kg/day, maximum 200 mg/d, titrated up every four weeks by 1 mg/kg/d after initiation at 0.5 mg/kg/d.
2. B-cell depletion (rituximab or biosimilar)
   1. Induction Dose: 750 mg/m2 (max 1000 mg) administered IV twice separated by two weeks.
   2. Maintenance Dose: 750 mg/m2 (max 1000 mg) administered IV once every six months thereafter.
3. Janus Kinase Inhibitors (tofacitinib or baricitinib)
   1. Tofacitinib: 5 mg orally twice per day.
   2. Baricitinib: 2 mg orally once daily.
4. Mycophenolate mofetil
   1. Goal dose of 1200 mg/m2/day divided twice daily, titrated up weekly by 300 mg/m2 intervals

The last dose of IVIg was administered two weeks prior to the start of any of the medications listed.

**Appendix B:** Determination of Abnormalities on Neurodiagnostic Studies

1. EEG: Focal or generalized slowing, focal epileptiform discharges out of any cortex, or seizure were considered abnormal. Generalized discharges were considered abnormal although inconsistent with the diagnosis of DSRD. All individuals had to have at least one prior EEG prior to the onset of DSRD symptoms that did not demonstrate these results.
2. Neuroimaging (MRI):
   1. T1: Abnormalities on T1 sequencing were defined as structural abnormalities of gray matter, white matter, brainstem, or cerebellum. Definition of hypoplasia of a structure was a region demonstrating volume loss but not structural defects and was assessed subjectively (quantitative volumetry not performed).
   2. T2: Abnormalities on T2/FLAIR sequencing were defined as lesions of gray matter or white matter measuring greater than 2 mm in diameter. Imaging abnormalities had to be present on both T2 and FLAIR images and had to be present on a two different field view (axial, sagittal, or coronal) to be defined as abnormal.
   3. SWI: Abnormalities on SWI sequences were defined as any signal abnormality of any size or distribution in the gray or white matter structures. Anatomical variants in the cerebrovasculature detected on SWI (e.g., hypoplastic vessel) were not considered abnormal unless they were deemed to be potentially clinically significant (e.g., narrowing of the internal carotid arteries consistent with moyamoya disease).
3. Abnormal Lumbar Puncture: Abnormalities were defined as having any of the following findings on a cerebrospinal fluid analysis:
   1. White blood cell (WBC) count > 5 cells/mm3
   2. Total protein > 60 mg/dL
   3. Presence of oligoclonal bands
   4. An immunoglobulin G (IgG) index of > 0.66,
   5. Neopterin elevation (> 33 nmol/mL).
      1. Samples with over 1,000 red blood cells (RBC) were excluded from analysis.
